# Book Notices

**Published:** 1895-05

**Authors:** 


### Book Notices.

TWENTIETH CENTURY PRACTICE. An International Encyclopedia of Modern Medical Science. By Leading Authors of Europe and America. Edited by Thomas L. Stedman, M. D., New York City. In Twenty Volumes. Volume II. Nutritive Disorders. New York: William Wood and Company. 1895.

Volume I. of this work was issued three months ago, and a notice of that volume, together with an outline of the plan and scope of the entire work, appeared in the March number of the JOURNAL. It is the purpose of the publishers to issue one volume every three months, until the whole series of twenty volumes have been published. That it will be the greatest medical work of this century, and a worthy successor of the great but too ancient Encyclopdæia of Ziemssen, we have no doubt.

In this volume, the following subjects are considered: Addison's Disease, and other Diseases of the Adrenal Bodies, by Sir Dyce Duckworth, M. D., LL. D., of London—31 pages.

Diabetes Mellitus, by Carl Von Noorden, M. D., of Frankfort-on-the-Main—154 pages.

Rheumatism, by T. J. Maclagan, M. D., of London—143 pages.

Gout, by Henry M. Lyman, M. D., of Chicago—182 pages.

Arthritis Deformous, by Archibald E. Garrod, M. D., of London—64 pages.

Diseases of the muscles, by G. S. Dujardin-Beaumetz, M. D., of Paris—48 pages.

Obesity, by M. J. Oertel, M. D., of Munich—103 pages.

The index covers twelve pages.

It will be seen that each subject is here allotted to a great medical man, and each one has contributed an exhaustive monograph on the subject assigned to him. The discussion of each disease, its history, causes, course, complications, morbid anatomy, etc., is so complete, the information here given on the subject is so full, that nothing seems to be lacking to make of this the ideal reference work. Some idea of the completeness of these articles can be gained by a perusal of the following subheadings used in Dr. Lyman's article on Gout:

Synonyms, history, definition, morbid anatomy, chemical characteristics of the tophi in gout, visceral lesions in gout, changes of the blood in gout, the urine in gout, symptomatology of gout, symptoms of acute gout, symptoms of chronic gout, chronic gout with visceral complications, irregular gout—abarticular gout, visceral gout affecting the digestive organs, hepatic changes, gouty affections of the circulatory apparatus, the respiratory organs, the nervous symptom, the influence of gout upon the genito-urinary organs, the influence of gout upon diseases of the skin, manifestations of gout in the organs of special sense, the morbid affinities of gout, the hereditary consequences of gout, the relations between gout and other intercurrent diseases. Etiology, geographical distribution of gout, climate, mode of life, heredity, age, sex, diet, bodily and mental exercise, season, poisons. Diagnosis. Prognosis. Pathology. Treatment, hygienic and prophylactic, diet, exercise, bathing medicinal treatment, alkalies, salicylic and benzoic acids, mineral acids, purgatives, mineral waters, treatment of acute gout, retrocedent gout, chronic gout, treatment of the obscure manifestations of the gouty diathesis, bibliography,

This volume contains a total of 739 pages, and is a handsome book.

H.

---

THE INTERNATIONAL MEDICAL ANNUAL AND PRACTITIONERS' INDEX. A work of Reference for Medical Practitioners. Thirty-seven Editors and Contributors. 648 pages. Illustrated. Thirteenth year—1895. Price in cloth. $2.75. E. B. Treat, Publisher, 5 Cooper Union, New York City.

"Treat's Annual" has been before the profession for the past twelve years, and the majority of American physicians are familiar with it. It furnishes valuable information not only concerning new remedies, but new applications of old ones. Whatever of real value to the profession has been contributed to medical literature during the year, through the medium of medical journals or otherwise, the new discoveries, new inventions, etc., is carefully noted in this volume and the main facts given. The greatest difficulty met with in the preparation of this volume, was to keep it within such reasonable limits as to render it a handiwork of reference without sacrificing its completeness.

The year 1894 witnessed many advancements in the science of medicine; a further study into the pathology and causation of disease has, in many instances, suggested a new line of therapeutics, and a further study into the physiological and therapeutical effects of certain drug has suggested new uses for them; some new remedies have been discovered, and some inventions in the way of appliances, etc., have been made during the past year, and all of these things have received their due consideration in the "Annual" for 1895.

Among the list of contributing American physicians to this volume we notice Drs. Allan McLane Hamilton, H. P. Loomis, John Ridlon, A. D. Rockwell, W. Blair Stewart, J. Madison Taylor, W. Gilman Thompson, W. B. Vanderpoel, Robert L. Watkins, Irving S. Haynes, and F. W. Koch.

Leading physicians of England, Scotland and other countries have assisted in the work of preparing this year's Annual. H.

---

We are in receipt of Frederick Stearns & Co.'s newly revised trade pamphlet on Wine and Cod-Liver Oil. This describes, in a concise manner, the theory relative to the active principles contained in cod-liver oil, on which they base their claim that the most effective method of administering cod-liver oil is by employing these active constituents of the oil from which the fatty matter has been removed in solution in a delicate wine. The results obtained from clinical work done with their preparation of Wine of Cod-Liver Oil, have been so uniformly favorable as to substantiate their claim that patients build up more rapidly, without becoming nauseated or suffering from digestive disturbances, on Wine of Cod-Liver Oil, than when the plain oil or emulsions of the oil are employed.

They have embodied in this little treatise nearly two hundred reports from physicians in active practice throughout the United States, and have compiled a therapeutic index, giving a list of ailments in which Wine of Cod-Liver Oil may be appropriately prescribed with benefit to the patient, and in most instances citing authorities who have employed it in these cases. Stearns' Wine of Cod-Liver Oil is a meritorious preparation, and should become known to every practitioner, for its efficacy, palatability, and ease of assimilation. It is a valuable aid to physicians who enploy cod-liver oil in their practice.

Messrs. F. Stearns & Co. will be pleased to forward a copy of treatise to any physician who will apply for it.

